# TTS2016R: A data set to study population and employment patterns from the 2016 Transportation Tomorrow Survey in the Greater Golden Horseshoe area, Ontario, Canada

**DOI:** 10.1177/23998083221146781

**Published:** 2023-01-09

**Authors:** Anastasia Soukhov, Antonio Páez

**Affiliations:** 3710 McMaster University, Hamilton, ON, Canada

**Keywords:** Jobs, population, work, commute, travel time, impedance, Greater Toronto and Hamilton Area, Greater Golden Horshoe area, Ontario, Canada, R

## Abstract

This paper describes and visualises the data contained within the {TTS2016R} data package created in R, the statistical computing and graphics language. {TTS2016R} contains home-to-work commute information for the Greater Golden Horseshoe area in Canada retrieved from the 2016 Transportation Tomorrow Survey (TTS). Included are all Traffic Analysis Zones (TAZ), the number of people who are employed full-time per TAZ, the number of jobs per TAZ, the count of origin destination (OD) pairs and trips by mode per origin TAZ, calculated car travel time from TAZ OD centroid pairs and associated spatial boundaries to link TAZ to the Canadian Census. To illustrate how this information can be analysed to understand patterns in commuting, we estimate a distance-decay curve (i.e. impedance function) for the region. {TTS2016R} is a growing open data product built on R infrastructure that allows for the immediate access of home-to-work commuting data alongside complimentary objects from different sources. The package will continue expanding with additions by the authors and the community at-large by requests in the future. {TTS2016R} can be freely explored and downloaded in the associated Github repository where the documentation and code involved in data creation, manipulation and all open data products are detailed.

## Introduction

This manuscript presents the open data product {TTS2016R}. Open data products are the result of turning source data (open or otherwise) into accessible information that adds value to the original inputs (see Arribas-Bel et al., 2021). The product presented in this paper is a R data package which consists of a fusion of objects from a variety of sources: home-to-work flows sourced from the 2016 Transportation Tomorrow Survey (TTS) (Data Management Group, 2018b), estimated travel times (calculated using {r5r} (Pereira et al., 2021)) and boundary files from the TTS (Data Management Group, 2018a) and from the Canadian Census (Statistics Canada, 2017).

What is a R data package? A R data package contains code, data and documentation in a standardised collection format that can be installed by R users through a centralized software repository such as CRAN (the Comprehensive R Archive Network) and GitHub. {TTS2016R} is available on GitHub for all to install and freely use in the spirit of open and reproducible research. Currently {TTS2016R} includes full-time home-based work-to-job origin destinations (OD) counts and mode-specific trip numbers retrieved from the 2016 TTS, traffic analysis zone (TAZ) boundaries, and municipality, planning and census metropolitan area boundaries for the Greater Golden Horse area (GGH) located in southern Ontario, Canada. In addition, the package includes TAZ centroid-to-centroid travel times by car, transit, cycling and walking mode computed using package {r5r} (Pereira et al., 2021).

The aim of this paper is to walk readers through the data sets, illustrate a use case (i.e. the calculation of an impedance function that can be used to calculate accessibility to employment) and invite others to experiment in its uses and applications. Though data from the TTS is freely available to the public through the TTS Data Retrieval System, the raw data can be technically demanding, cumbersome to work with and requires multiple software to process. By pre-processing the data, packaging it with complimentary data and providing explicit documentation in a R environment, {TTS2016R} offers a slice of the TTS data that can be immediately used by R users to analysis patterns of commuting to work in the region. Anticipate this package to grow in the future: it currently provides an open infrastructure for additional TTS or complimentary data sets to be amended by the authors and the open-source community in the future by request.

### Home-to-work commute data

**Figure 1.**
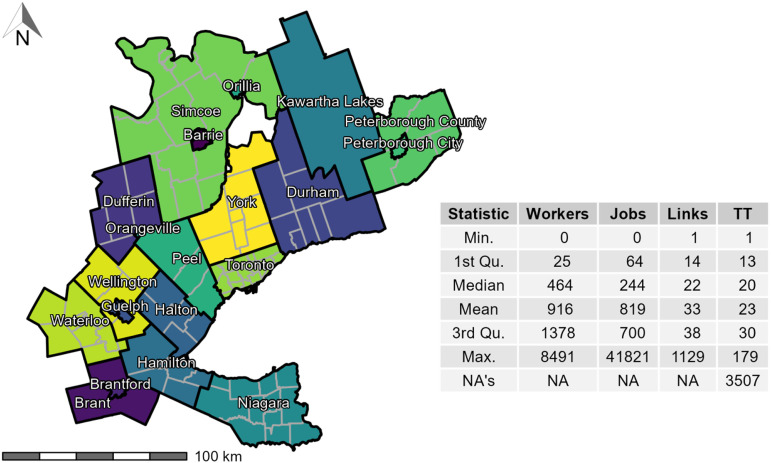
TTS 2016 study area within the GGH in Ontario, Canada, along with associated descriptive statistic of workers and jobs per TAZ, OD links (count of workers potentially interacting with their place of employment) by origin TAZ, and calculated OD car travel time (TT) per origin TAZ. 3507 trips were not assigned TT as they are longer than 180 mins. Spatial boundary files are retrieved from the TTS which define the survey area (Data Management Group, 2018a): the 20 regions in the GGH are represented by black lines and labelled, the dark grey lines are planning boundaries.

Currently, {TTS2016R} includes counts of full-time employed population by place of residence (origin), counts of full-time usual place of work (destination), number of trips to work by mode and the calculated potential travel time of the trips in the GGH. The GGH (and hence the TTS survey area) is displayed in Figure 1.

This data is aggregated and available at the level of TAZ: TAZ are a spatial unit of analysis typically used to estimate the number of trips produced and attracted to each zone (Meyer and Miller, 2001). They are thus defined by transportation planners for a region based on intra-similarity and inter-dissimilarity between land-use and population demographics. Within the GGH boundaries, 3764 TAZ are specified and each TAZ is uniquely identified using the GTA06 Zoning System: the survey boundary is discussed in the 2016 TTS methodology and defined by the TTS (Data Management Group, 2018b). The TAZ range between ≥ 0.019 km^2^ in spatial area to a maximum of 879 km^2^ (median: 1.3 km^2^ and 3rd quantile: 2.8 km^2^).

### Full-time employed people and associated places of employment

In the GGH, there are 3,446,957 workers (i.e., residential origins), 3,081,900 jobs and 3,282,611 work-related trips (for the 2016 TTS survey day). The values are organized within the origin destination (OD) table in the {TTS2016R} package and are derived from the cross-tabulation by person and by trip for the full-time employed population and associated places of employment.

The total number of full-time workers and jobs in the 2016 TTS region are not equal. Since the outer boundaries of the TTS are permeable, workers who reside within the boundaries but have workplaces that are outside of the boundaries are counted as workers within an origin TAZ, while jobs in TAZ that are filled by workers who reside outside the GGH boundaries are *unknown* since they were not surveyed. This mismatch results in the total number of workers being 1.12 times larger than the number of jobs. The TTS is a proportionally representative survey; hence, the values included in {TTS2016R} are adjusted to reflect the GGH working population and their home-based trips to places of GGH employment.

The count of links and trips made by the full-time working population and associated full-time place of employment per unique OD pair are quite variable. TAZ contain between 0 and 8491 workers (median: 464, 3rd quantile: 1378), 0 to 41,821 jobs (median: 244, 3rd quantile: 700), and generate between 0 to 241 trips (median: 15, 3rd quantile: 42).

**Figure 2.**
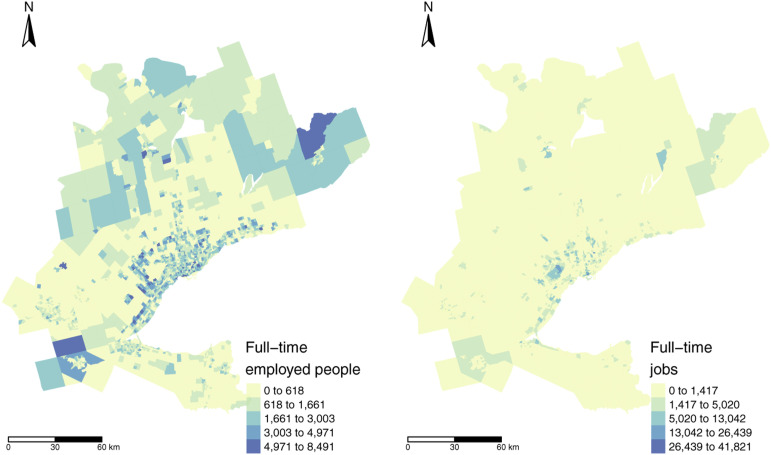
Number of workers (left) and jobs (right) in each TAZ retrieved from the 2016 TTS (Data Management Group, 2018b). Spatial boundary files are retrieved from the TAZ defined by the TTS (Data Management Group, 2018a).

Figure 2 presents the number of employed people and associated jobs per TAZ. It can be observed that the spatial distribution of jobs and workers is unequal, which is indicative of jobs – housing imbalance that can have an impact on accessibility in a region (Levine, 1998). There is also a higher number of TAZ with no workers than zones with no jobs (i.e. 791 TAZ with no workers and 396 TAZ with no jobs) and the mean of workers per TAZ is higher than the mean of jobs. The number of TAZ with an extreme number of jobs at the highest and lowest percentiles is significantly higher than the number of workers.

### Calculated travel time

**Figure 3.**
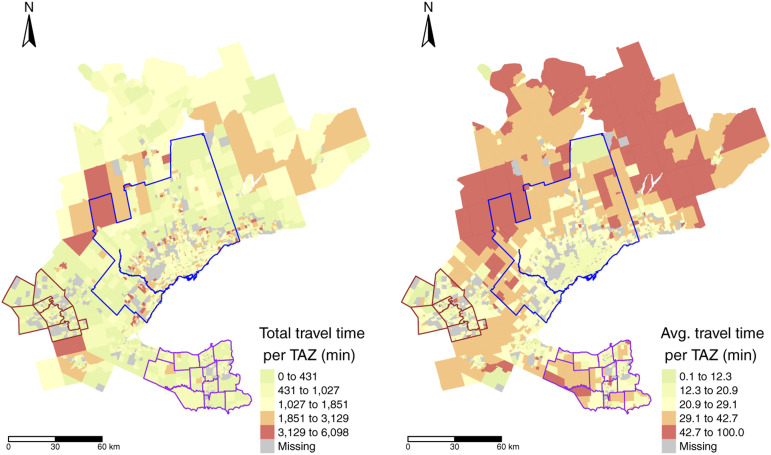
Calculated total worker travel time by car (left) and average worker travel time by car (right) for each TAZ in the 2016 TTS. Car travel times are calculated using {r5r} (Pereira et al., 2021) assuming posted road network travel speeds. Planning boundaries of Niagara and Waterloo (Data Management Group, 2018a), and the Toronto census metropolitan area (Statistics Canada, 2017) are drawn with purple, brown and blue borders, respectively.

Also included in {TTS2016R} are the estimated travel times between OD as summarized in the descriptive statistics table in Figure 3; travel times are calculated using the package {r5r}. {r5r} interfaces with the java-based R5 routing engine developed separately by Conveyal (Conveyal, 2022). The inputs to {r5r} for this data package were: the desired mode, a maximum travel time threshold of 180 minutes, the geo-coded origin destination pairs based on the centroids of the TAZ and the static OpenStreetMap road network of Ontario (retrieved using Geofabrick (Geofabrik, 2022)). A travel time threshold of 180 minutes was selected since it captures almost all potential OD interactions.

Additionally, car travel is included in this data package since it is a critically important commute mode in the GGH. 2,598,379 of the trips are made using a car out of the total 3,282,611 work-related trips according to the TTS 2016 data (i.e., 79% of trips are taken by car).

These travel times are a useful addition to {TTS2016R} since they are not included in the TTS Data Retrieval System but they are vitally important to estimate the cost of travel and associated impedance functions, among other possible applications. If the readership is interested in additional information regarding the travel time computation, please see the calculation notebook in the documentation of {TTS2016R} and details about {r5r} at the package website.

As can be observed in Figure 3, the total travel time resembles the spatial trend distribution in the number of employed people in the previous plot (Figure 2) and the spatial distribution of the average travel time is distinct from other plots presented so far. We can see that in areas around the south-eastern border such as Niagara and Waterloo (purple and brown borders), the average travel times are moderately low. Additionally, travel times (by car) within the core of the Toronto census metropolitan area (CMA) (blue) is also moderately low since traffic congestion is not reflected in the travel time calculations. Further from these areas, travel times are higher.

### Calibrating an impedance function

An application of the {TTS2016R} package is the calculation of an impedance function. Impedance functions are useful to understand mobility behaviour and are used to estimate gravity models of spatial interaction (Haynes and Fotheringham, 1985; Wilson, 1971) and applied in accessibility analysis (Barboza et al., 2021; Hansen, 1959; Páez et al., 2013; Talen and Anselin, 1998). An impedance function *f*(⋅) depends on the cost of travel *c*_*ij*_ between locations *i* and *j* (all which is supplied in the travel time and origin destination table within {TTS2016R}).

A useful technique to calibrate an impedance function is to use the trip length distribution (TLD) as measured from OD data (Batista et al., 2019; Horbachov and Svichynskyi, 2018). The TLD is the representation of the likelihood that a proportion of trips are taken at a specific travel cost. In our data set, where we assume cost is travel time, the impedance function maps low travel times to higher proportions of trips, and high travel times are mapped to low proportion of trips.

**Figure 4.**
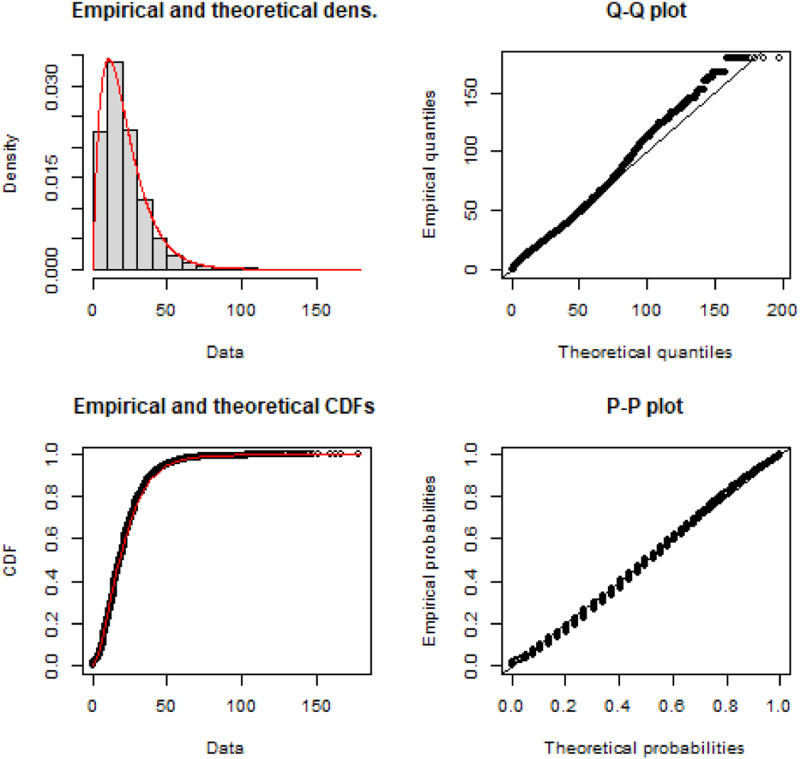
Empirical TTS 2016 home-based car TLD (black) and calibrated gamma distribution impedance function (red) with associated Q-Q and P-P plots.

Using the data contained in {TTS2016R}, we fit the empirical TLD to a density distribution using maximum likelihood techniques and the Nelder-Mead method for direct optimization available within the R package {fitdistrplus} (Delignette-Muller and Dutang, 2015). Based on goodness-of-fit criteria and diagnostics seen in Figure 4, the gamma distribution is selected. The ‘shape’ parameter is *α* = 2.019, the estimated ‘rate’ is *β* = 0.094 and Γ(*α*) is defined in equation (1).

(1) $$\begin{array}{l} f\left(x,\alpha ,\beta \right)=\frac{x^{\alpha -1}e^{-\frac{x}{\beta }}}{\beta^{\alpha }\Gamma \left(\alpha \right)}\;\text{for}\;0\leq x\leq \infty \\ \Gamma \left(\alpha \right)=\int_{0}^{\infty }x^{\alpha -1}e^{-x}\;dx \end{array}$$

## Concluding remarks

This paper introduces {TTS2016R}, an open data product in the form of a R data package. This package is a fusion of data from multiple sources and we demonstrate the spatial and numeric extent of the data contained within. It includes an OD cross-tabulation by person and by trip mode table for home-to-work commute data from the 2016 TTS alongside complimentary boundaries and estimated travel times. The value of this data package is in its transparency, easy of access and its open infrastructure for the addition of complimentary data sets in the future. R users can immediately and easily explore GGH commute flow trends as well as suggestion further amendments to the package by request. One possible use of this data, as showcased in this paper, is the calibration of impedance functions which in turn can be used for accessibility analysis.

In the spirit of novel and original research, we hope readers value the efforts made to detail the data in order to improve transparency in our work and encourage others to replicate and, hopefully, inspire work of their own. We see this product as providing open infrastructure for additional TTS or complimentary data sets to be amended by the authors or wider open-source community in the future.
